# Prevalence and Clinical Correlates of Sleep Disorders in RFC1‐Spectrum Disorders: A Cross‐Sectional Study

**DOI:** 10.1002/mds.30279

**Published:** 2025-07-21

**Authors:** Antonio Funcis, Valerio Brunetti, Salvatore Rossi, Giammarco Dalla Zanna, Martina de Scisciolo, Giulia Maneri, Enrico Di Stasio, Antonio Petrucci, Giacomo Della Marca, Gabriella Silvestri

**Affiliations:** ^1^ Department of Neurosciences Università Cattolica del Sacro Cuore Rome Italy; ^2^ UOC di Neurologia – Dipartimento di Neuroscienze, Organi di Senso e Torace Fondazione Policlinico Universitario Agostino Gemelli IRCCS Rome Italy; ^3^ Departmental Unit of Molecular and Genomic Diagnostics, Genomics Core Facility Gemelli Science and Technology Park (G‐STeP), Fondazione Policlinico Universitario Agostino Gemelli IRCCS Rome Italy; ^4^ Department of Basic Biotechnological Sciences, Intensive Care and Perioperative Clinics Università Cattolica del Sacro Cuore Rome Italy; ^5^ Dipartimento di scienze laboratoristiche ed infettivologiche, UOC Chimica, Biochimica e biologia molecolare clinica Fondazione Policlinico Universitario Agostino Gemelli IRCCS Rome Italy; ^6^ Center for Neuromusculr and neurological rare diseases S.Camillo Forlanini Hospital Rome Italy

**Keywords:** CANVAS, cerebellum, inherited ataxia, RFC1, sleep disorder

## Abstract

**Background:**

Sleep disturbances in RFC1‐spectrum disorders remain unexplored. This study aimed to investigate the prevalence and characteristics of sleep disorders in patients with RFC1‐spectrum disorders and their impact on quality of life (QoL).

**Methods:**

Patients underwent a comprehensive sleep assessment using validated questionnaires, polysomnography, and QoL evaluation.

**Results:**

Among 16 participants, 15 (94%) had obstructive sleep apnea: severe in four, moderate in nine, and mild in two. Restless legs syndrome affected 12 patients (75%), with moderate‐to‐severe symptoms in 11. Periodic limb movements during sleep exceeded the clinical threshold in 10 patients (63%). Rapid eye movement (REM) sleep behavior disorder was identified in 1 patient. Subjective assessments revealed poor sleep quality in 11 patients (69%) and insomnia in 10 (63%). All patients exhibited reduced QoL. Reduction in slow‐wave sleep and REM sleep are associated with increased clinical severity.

**Conclusion:**

RFC1‐spectrum disorders are associated with a high prevalence of sleep disorders and impaired QoL, highlighting the need for screening. © 2025 The Author(s). *Movement Disorders* published by Wiley Periodicals LLC on behalf of International Parkinson and Movement Disorder Society.

## Graphical abstract

The RFC1‐spectrum disorders include multisystem manifestations that extend beyond cerebellar ataxia, neuropathy, and vestibular areflexia syndrome (CANVAS). Sleep disorders constitute a significant component of the disease's non‐motor spectrum. Obstructive sleep apnea, restless legs syndrome, periodic limb movements of sleep, and insomnia are common in RFC1‐related disease and should be actively investigated.

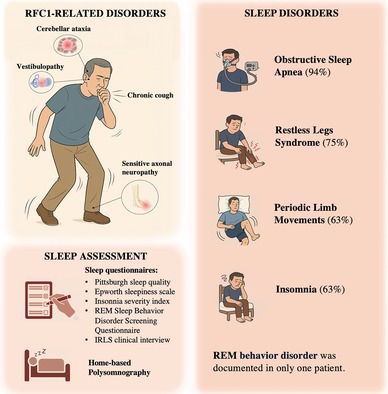

The RFC1‐spectrum disorders have expanded to include multisystem manifestations that extend beyond the triad of cerebellar ataxia, neuropathy, and vestibular areflexia syndrome (CANVAS), involving motor and non‐motor systems.^1^ To date, no studies have characterized sleep disorders in such conditions. This study aimed to evaluate the prevalence of sleep disorders in patients with RFC1‐spectrum disorders and to assess their impact on quality of life (QoL).

## 1 Methods

This was an observational study with a cross‐sectional design conducted from March 2023 to June 2024. We recruited 16 patients with genetically confirmed RFC1 biallelic n(AAGGG) expansions.^2^ Participants provided written informed consent, and the study was approved by the local ethics committee. We followed the Strengthening the Reporting of Observational Studies in Epidemiology (STROBE) reporting guidelines.^3^ The primary outcome measures were the prevalence of sleep‐disordered breathing, restless legs syndrome (RLS), rapid eye movement (REM) sleep behavior disorder (RBD), and insomnia.

### 1.1 Clinical, Radiological Data, and Genetic Analysis

The techniques for genetic diagnosis and collection of clinical data are detailed in Data S1. Clinical assessment included evaluation of motor impairment using the Scale for the Assessment and Rating of Ataxia (SARA), health‐related quality of life (HRQoL) using the 36‐item Short Form Health Survey (SF‐36), and anxiety and depressive symptoms using the Zung Self‐Rating Anxiety Scale (SAS) and the Beck Depression Inventory‐Short Form (BDI‐SF), respectively. All patients underwent brain magnetic resonance imaging (MRI) at 1.5 Tesla (T).

### 1.2 Sleep Assessment

Sleep was evaluated using self‐administered questionnaires and home‐based polysomnography (PSG) described in Data S2. Sleep quality was assessed using the Pittsburgh Sleep Quality Index (PSQI),^4^ excessive daytime sleepiness (EDS) was measured with the Epworth Sleepiness Scale (ESS),^5^ and insomnia by the Insomnia Severity Index (ISI).^6^ RLS and periodic limb movements of sleep (PLMS) were diagnosed according to standard criteria.^7^, ^8^ Patients and their bed partners were interviewed separately about RBD using the RBD Screening Questionnaire (RBDSQ).^9^ Sleep stages and sleep‐related events were scored by a certified sleep physician (V.B.).^10^ REM without atonia (RWA) was defined according to the Montreal method.^11^

### 1.3 Statistical Analysis

Continuous and categorical variables were summarized as mean and standard deviation (SD) and count and percentage, respectively. Spearman's rho correlation coefficients were computed between the demographic data, SARA score, disease duration, HRQoL, and sleep measures from the PSG and questionnaires. After Bonferroni correction for multiple comparisons, the *P*‐value was set at <0.0005. To measure the strength of the relationship, the effect size (ie, independent of sample size) was calculated and converted into four classes according to the r metric (ie, <0.1 = very small; 0.1–0.3 = small; 0.3–0.5 = medium; >0.5 = large) and only r > 0.5 (large effect) were considered relevant to avoid overinterpretation. All statistics were performed using SPSS v.28.

## 2 Results

### 2.1 Demographic, Clinical, Radiological and QoL Characteristics

We enrolled 16 patients (9 males, 69%). Mean age was 64 ± 6 years, with a mean disease duration of 15 ± 6 years and SARA score of 6 ± 5. Sensory axonal polyneuropathy and chronic cough were present in all patients, while cerebellar ataxia was present in 14 patients. CANVAS was the clinical presentation in 12 patients. Dysautonomia and vestibulopathy were present in 10 and 13 patients, respectively. Five patients reported mild dysphagia. According to the SAS and BDI‐SF, anxious and depressive symptoms were present in 6 and 4 patients, respectively. Detailed HRQoL measurements are provided in Data S3. Brain MRI data are described in Data S4. Full demographic, clinical, and radiological characteristics are shown in Table S1.

### 2.2 Sleep Questionnaires

Eleven patients (69%) complained of poor sleep quality according to the PSQI. Insomnia was present in 10 patients (63%), of whom 6 were moderate‐to‐severe. Eleven patients (69%) reported using sedative‐hypnotics, including 3 patients (19%) for insomnia, 7 (44%) patients for neuropathic discomfort, and 1 patient (6%) for depression (details in Data S5). Two patients (13%) complained of EDS. Twelve patients (75%) met the clinical diagnostic criteria for RLS, with a mean severity of 14 ± 10 on the International Restless Legs Syndrome Study Group (IRLSSG) score, and 11 suffered from moderate‐to‐very‐severe RLS. Ferritin levels were above 100 ng/mL in all patients. Based on the RBDSQ, 6 patients (38%) had symptoms suggestive of RBD. Detailed sleep questionnaires are shown in Table 1.

**Table 1:** Sleep measures in patients with RFC1‐spectrum disorders.

Sleep measure	Patients (N = 16)	
	Mean (SD)	n (%)
Sleep questionnaires		
PSQI (score > 5)	8 (4)	11 (69)
ESS (score > 10)	8 (6)	2 (13)
ISI (score > 8)	12 (6)	10 (63)
Clinical RLS		12 (75)
RBDSQ (score > 4)	4 (2)	6 (38)
Polysomnography data		
Night‐time sleep		
Time in bed (min)	412 (70)	
TST (min)	365 (69)	
Sleep efficiency (%)	83 (10)	
Sleep latency (min)	15 (19)	
Sleep stages (%)		
N1/TST	12 (7)	
N2/TST	50 (13)	
N3/TST	21 (13)	
REM/TST	17 (6)	
Sleep fragmentation		
Awakenings >1 min (n)	8 (6)	
Wake after sleep onset (min)	59 (47)	
Respiratory parameters		
AHI total (events/h)	23 (13)	
Obstructive AHI (events/h)	22 (14)	15 (94)
Central AHI (events/h)	1 (2)	1 (6)
ODI (events/h)	21 (12)	
T90 (%)	7 (16)	
Mean saturation in sleep (%)	94 (2)	
Lowest saturation in sleep (%)	79 (8)	
Periodic limb movements		
PLMSi (events/h)	37 (39)	10 (63)
REM without atonia		1 (6)
Phasic RWA (% of REM sleep time)	6 (14)	
Tonic RWA (% of REM sleep time)	4 (11)	

*Note*: The second column shows the mean (SD) and the third column shows the number (%) of patients above the cut‐off.

Abbreviations: SD, standard deviation; PSQI, Pittsburgh Sleep Quality Index; ESS, Epworth Sleepiness Scale; ISI, Insomnia Severity Index; RLS, restless leg syndrome; RBDSQ, REM Sleep Behavior Disorder Screening Questionnaire; TST, total sleep time; REM, rapid eye movement; AHI, Apnea‐Hypopnea Index; ODI, Oxygen Desaturation Index; T90, percentage of sleep time with oxygen saturation < 90%; PLMSi, Periodic Limb Movement of Sleep Index; RWA, REM without atonia.

### 2.3 Polysomnography

Detailed PSG data are listed in Table 1. OSA was identified in 15 patients (94%), with 1 patient exhibiting a mixed form of both obstructive and central sleep apnea. OSA was severe in 4 patients, moderate in 9 patients, and mild in 2 patients. Patients with moderate‐to‐severe OSA had higher ESS scores compared with patients without OSA (OSA: 8 ± 5 vs 5 ± 5). PSG confirmed the diagnosis of RBD in 1 patient who was being treated with clomipramine for depression. Ten patients (63%) showed a PLMS index >15 events/hour. Detailed PSG data are listed in Table 1.

### 2.4 Statistical Correlations

**Figure 1:**
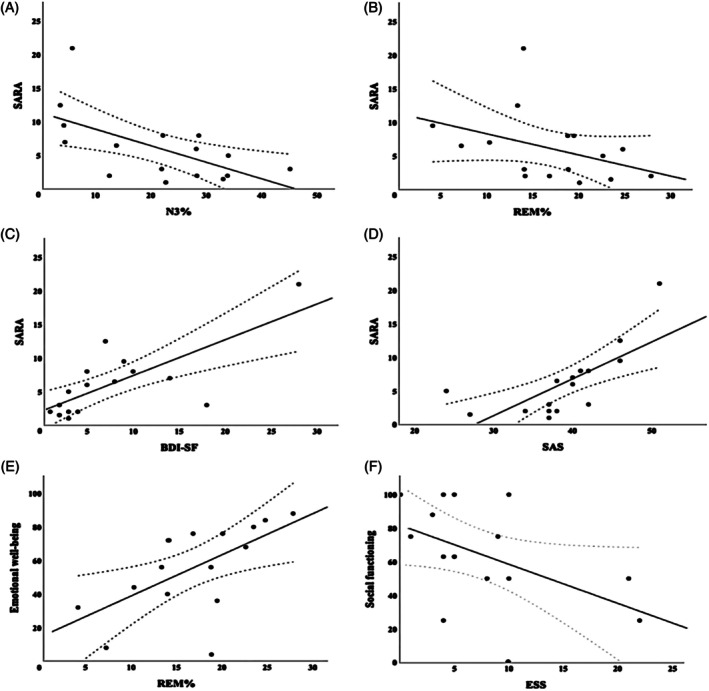
Scatter plots with linear regression fit and 95% confidence interval of the most relevant significant correlations. (A, B) Negative correlation between Scale for the Assessment and Rating of Ataxia (SARA) score with N3% and REM%. (C, D) Positive correlation between SARA score with Beck Depression Inventory‐Short Form (BDI‐SF) and Zung Self‐Rating Anxiety Scale (SAS). (E) Positive correlation between REM% and Emotional well‐being. (F) Negative correlation between Epworth Sleepiness Scale (ESS) and Social functioning.

Due to the intrinsic low number of cases for a rare disease, none of correlations performed reached the statistical significance of *P* < 0.0005. However, analysis of the effect size revealed a strong negative association between SARA and several HRQoL parameters (General health: ρ = −0.634, *P* = 0.008; Bodily pain: ρ = −0.723, *P* = 0.002; Social functioning: ρ = −0.709, *P* = 0.002; Vitality: ρ = −0.518, *P* = 0.040; Emotional problems: ρ = −0.585, *P* = 0.017; Physical problems: ρ = −0,605, *P* = 0.013; Physical functioning: ρ = −0.765, *P* = 0.001), and a positive correlation between SARA and BDI‐SF (ρ = 0.721, *P* = 0.002) and SAS (ρ = 0.808, *P* = 0.001). Higher SARA was associated with a lower N3 (ρ = −0.566, p = 0.022) and REM sleep (ρ = −0.567, *P* = 0.022). We found that the higher REM was associated with higher values of QoL (Social functioning: ρ = 0.528, *P* = 0.035; Emotional well‐being: ρ = 0.666, *P* = 0.005; Vitality: ρ = 0.617, *P* = 0.011; Physical problems: ρ = 0.659, *P* = 0.006; Physical functioning: ρ = 0.520, *P* = 0.039), while daytime sleepiness was associated with lower values of QoL (Bodily pain: ρ = −0.562, *P* = 0.024; Social functioning: ρ = −0.508, *P* = 0.045; Emotional well‐being: ρ = −0.519, *P* = 0.039; Vitality: ρ = 0.571, *P* = 0.021). Relevant correlations are shown in Figure 1.

## 3 Discussion

Our findings demonstrate that OSA, RLS, and insomnia are highly prevalent among patients with RFC1‐related disorders when actively investigated. The prevalence was significantly higher compared with previous reports of CANVAS patients.^12^, ^13^ OSA is the most frequent sleep disorder, identified in nearly all patients (94%), and with the majority exhibiting moderate‐to‐severe OSA. Although these patients require continuous positive airway pressure therapy, 5 patients were unable to tolerate it due to an uncontrollable cough reflex. These patients present a therapeutic challenge and may require alternative treatments, such as mandibular advancement devices.

OSA is a well‐established risk factor for vascular diseases^14^, ^15^ and may play a role as a risk factor for neurodegenerative diseases.^16^ Consequently, OSA may partly contribute to neurodegeneration in patients with RFC1‐related disorders.

OSA has an estimated prevalence ranging from 9% to 38% in the general population.^17^ It is important to emphasize that none of our patients exhibit known risk factors (eg, obesity or increased neck circumference) that would explain such a high frequency of this comorbidity.^18^ Its high prevalence may be related to the associated autonomic and peripheral nervous system degeneration. We may hypothesize that OSA and chronic cough, a core symptom of RFC1‐related disorders, share a common pathophysiological origin driven by dysfunction of the vagus nerve. Neuronal loss in the vagal nuclei,^19^ as well as dysfunction of vagal sensory pathways, can impair the control of pharyngeal muscle tone, which is essential for maintaining upper airway patency during sleep, and may contribute to an exaggerated cough reflex.^20^ OSA and chronic cough have been observed in patients undergoing vagal nerve stimulation.^21^

Notably, only 2 patients with OSA complained of EDS. The low prevalence of subjective EDS may reflect a chronic adaptation to longstanding sleep fragmentation or may be influenced by concomitant cognitive impairment associated with RFC1‐related disorders, potentially reducing awareness and perception of EDS.^22^ Although a few patients met the criteria for EDS, we observed that as sleepiness increased, physical and mental QoL measures worsened.

We observed a high prevalence of RLS in patients with RFC1‐related disorders, with 75% of patients exhibiting moderate‐to‐severe forms. Moreover, there was a high frequency of PLMS in our population, a condition often associated with both RLS and OSA.^23^ All patients reported circadian variation in symptoms being prevalent during the evening and at night. In our cohort, RLS, rather than idiopathic, is likely secondary to underlying neuropathy.^24^ In fact, all our patients presented with sensory axonal neuropathy, and it is well established that RFC1‐related diseases are characterized by severe and widespread sensory neuropathy also affecting small nerve fibers, that have been implied in the pathogenesis of RLS.^25^ Additionally, dysfunction in the dopaminergic pathway, previously reported in CANVAS/RFC1‐related disorder,^26^ may contribute to RLS.

RWA was documented in 1 patient, despite 6 patients reporting symptoms suggestive of RBD. Notably, the patient with confirmed RBD was taking a tricyclic antidepressant, which is well known to promote RBD; therefore, this case should be considered as secondary RBD due to antidepressant use.^27^ It is well‐established that RBD screening questionnaires have low specificity and low positive predictive compared with PSG.^28^ This discrepancy suggests that caregivers/neurologists may misinterpret certain symptoms, such as PLMS or arousals induced by OSA, with RBD‐like behavior. Thus, PSG may be helpful for differentiating RFC1‐spectrum disease from other late‐onset ataxia disorders. In fact, the low frequency of RBD and high prevalence of OSA observed in our population contrast with multiple systemic atrophy type C, in which RBD and nocturnal stridor often coexist with both obstructive and central apneas,^29^ and with spinocerebellar ataxia type 3, in which RLS and RBD are the most prevalent sleep disorders.^30^

Moreover, we observed significant impairments in sleep quality, as reflected by high PSQI and ISI scores. PSGs showed reduced total sleep time and increased wake after sleep onset relative to expert sleep recommendations.^31^ The pathophysiology of insomnia in these patients is likely multifactorial. The high prevalence of comorbid sleep disorders, such as OSA and RLS, may contribute to fragmented sleep. Furthermore, anxiety and depressive symptoms, both highly prevalent in our cohort, are well‐established contributors to the development of insomnia.^32^

The QoL in our cohort showed a low perception of general health, with a predominant impairment in motor function. Limitations in daily activities were primarily due to motor problems, pain, and fatigue. Emotional well‐being was reduced, though social interactions were preserved.

Reduced REM and N3 sleep were associated with greater motor impairment and lower mental and physical QoL, though older age may confound these associations by correlating with both disease severity and diminished REM and N3 sleep. Overall, motor impairment, sleep disturbances, and reduced QoL may exert reciprocal negative influences, collectively contributing to affecting the well‐being in patients with RFC1‐related disorders.

A key limitation of our study is its single‐center design and relatively small sample size, reflecting the rarity of the disease. Additionally, a significant proportion of patients were taking medications that can influence sleep. Finally, correlation analysis cannot establish a causal relationship between the variables examined, and the limited sample size of the study precludes the drawing of definitive conclusions. However, this study represents the first comprehensive investigation into sleep disturbances in RFC1‐related disorders.

## 4 Conclusions

RFC1‐related disease is characterized by a high prevalence of sleep disorders and poor QoL, which constitute a significant component of the disease's non‐motor spectrum. OSA, RLS, and insomnia are the most common sleep disorders in RFC1 pathology. The broad array of sleep disturbances observed emphasizes the importance of systematic screening protocols.

## Author Roles

(1) Research Project: A. Conception, B. Organization, C. Execution; (2) Statistical Analysis: A. Design, B. Execution, C. Review and Critique; (3) Manuscript Preparation: A. Writing of the First Draft, B. Review and Critique.

A.F.: 1A, 1B, 1C, 2A, 2B, 3A.

V.B.: 1A, 1B, 1C, 2A, 2B, 3B.

S.R.: 1C, 3B.

G.D.Z.: 1C.

M.d.S.: 1C.

G.M.: 1C, 2B.

E.D.S.: 2A, 2B, 2C.

A.P.: 1C.

G.D.M.: 3B.

G.S.: 1A, 3B.

## Financial Disclosures

None of the authors report any financial disclosures.

## Supporting information


Data S1

## Data Availability

The data that support the findings of this study are available from the corresponding author upon reasonable request.
