# Supplementary material for: Prevalence and Clinical Correlates of Sleep Disorders in RFC1‐Spectrum Disorders: A Cross‐Sectional Study
Source: Mov Disord. 2025 Jul 21;40(9):1990–5. doi: 10.1002/mds.30279 (PMC12485576; doi:10.1002/mds.30279)
Supplement: Supplementary file 1 — Data S1 [file MDS-40-1990-s001.docx]

**Prevalence and clinical correlates of sleep disorders in RFC1-spectrum disorders: a cross-sectional study**

Supplementary Data S1

*Inclusion-Exclusion Criteria, Clinical-radiological Data and Genetic Analysis*

We screened all patients with a genetically confirmed RFC1 biallelic n(AAGGG) expansions^1^ followed at our center. Exclusion criteria were: 1) inability to undergo polysomnography due to advanced disease-related disability, 2) respiratory, cardiac, or renal failure, and 3) active cancer. We enrolled 16 patients. Two patients were excluded because they were bedridden, and one was undergoing chemotherapy for breast cancer. All patients underwent to a neurological examination and sleep assessment by certified physicians.

Demographic and clinical data, comorbidities, and the use of sedative-hypnotics were collected. The age of onset was defined as the onset of ataxia or sensory neuropathy. The Scale for the Assessment and Scoring of Ataxia (SARA) was used to estimate the extent of motor impairment^2^. These data were collected from medical records and then confirmed during a face-to-face interview conducted at follow-up to avoid bias. The measurement of health-related quality of life (HRQoL) was analyzed by means of the 36-item Short Form Health Survey (SF-36). The SF-36 is a self-administered questionnaire and consists of two main domains: the physical component comprising physical functioning (PF), role limitations due to physical problems (RP), bodily pain (BP), perception of general health (GH), and the mental component comprising vitality (VT), social functioning (SF), role limitations due to emotional health problems (RE), emotional well-being (EW). Scores range from 0 to 100, with higher scores indicating better physical and mental well-being^3^. Symptoms of anxiety and depression were evaluated using the Zung Self-Rating Anxiety Scale (SAS), with a raw cut-off score>40 indicating presence of clinical anxiety^4^, and the Beck Depression Inventory-Short Form (BDI-SF), with a raw cut-off score>13 indicating presence of symptoms of depression^5^, respectively.

Vestibulopathy was defined in all patients by vestibular assessment using VHINT (Video Head Impulse Test) and videonystagmography, conducted by ORL specialist. Dysautonomia was assessed by face-to-face interview using the COMPASS-31 questionnaire. All patients underwent brain magnetic resonance imaging (MRI) scan at 1.5 Tesla (T). The following sequences were acquired: T1-weighted, T2-weighted and fluid-attenuated inversion recovery (FLAIR) sequence. All images were evaluated by a certified neuroradiologist.

Genomic DNA isolated from peripheral blood leucocytes was used to screen for biallelic intronic expansions (AAGGG)exp in intron 2 of the RFC1 gene. First, a SHORT-RANGE flanking PCR was performed to test for the presence or absence of the amplified region. In the absence of AAGGG pathogenetic amplification, repeat-primed PCR (RP-PCR) was performed to confirm affected individuals. All primers were the same as those used by Cortese et al. (2019)^1^.

Supplementary Data S2

*Sleep questionnaires*

Sleep was evaluated using self-administered questionnaires and home-based polysomnography (PSG). Sleep quality was assessed using the Pittsburgh Sleep Quality Index (PSQI), where a score >5 indicates poor sleep quality^6^. Daytime sleepiness was measured with the Epworth Sleepiness Scale (ESS), with a cut-off score of >10 indicating excessive daytime sleepiness^7^. Insomnia symptoms were assessed during a face-to-face interview and its severity was evaluated using the Insomnia Severity Index (ISI), and categorized based on the severity of insomnia (mild: 8<ISI<14, moderate: 15≤ISI<21, and severe: ISI >21)^8^. The presence of Restless Legs Syndrome (RLS) was diagnosed according to standard criteria during a face-to-face interview^9^ and further confirmed by certified sleep physician (V.B.) to exclude mimics. For individuals with consistent symptoms, RLS severity was assessed using the International RLS Study Group Rating Scale (IRLSGGS) and classified as mild (IRLSGGS≤10), moderate (10<IRLSGGS≤20), severe (20<IRLSGGS≤30), and very severe (IRLSGGS>30)^10^. Diagnostic assessment included determination of serum ferritin levels in all patients, to rule out the presence of concomitant iron deficiency in relation to RLS.

Additionally, patients and their bed partners were separately interviewed about rapid eye movement (REM) sleep behavior disorder (RBD) during the current year using REM Sleep Behavior Disorder Screening Questionnaire (RBDSQ), with cut-oft score of >4^11^. RBD was further confirmed by means of detection of REM Sleep Without Atonia (RWA) on the PSG.

*Polysomnography*

The monitoring includes 8 EEG leads (Fp1, Fp2, T3, T4, C3, C4, O1, O2) referred to contralateral mastoid; right and left electro-oculogram; oro-nasal airflow; snoring; thoracic and abdominal efforts; ECG; pulse oximetry; and submentalis and tibialis anterior EMG. Sleep stages, movements during sleep and sleep-related respiratory events were manually scored according American Academy of Sleep Medicine criteria^12^ by a certified sleep physician (V.B.). RWA was defined according to the Montreal method^13^. Obstructive sleep apnea (OSA) was identified by an obstructive apnea-hypopnea index (AHI)>5 events/hour and classified according to the AHI in mild (AHI < 15 events/hour), moderate (15<AHI<30 events/hour) and severe (AHI>30 events/hour), while central sleep apnea (CSA) was defined by a central AHI>5 events/hour. The Periodic Limb Movements of Sleep Index (PLMSi) was defined as the number of periodic limb movements meeting PLMS criteria per hour of sleep^14^; notably, PLM associated to respiratory events were included in the count of the PLMSi. A cut-off value of PLMSi>15 events/hour was used to identify patients with an elevated frequency of PLMS.

The diagnosis of a specific sleep disorder was further confirmed by a certified sleep physician (V.B.) following the International Classification of Sleep Disorders Third Edition^14^.

Supplementary Data S3

*Quality of Life measurements*

All patients reported a low perception of general health. Regarding the physical health component, the patients exhibited impaired physical functioning (mean = 60, SD = 31), with significant limitations in daily activities due to physical problems (mean = 39, SD = 41) and bodily pain (mean = 58, SD = 31). In terms of the mental health component, the patients reported increased fatigue (mean = 48, SD = 24), and decreased emotional well-being (mean = 56, SD = 26). Although emotional problems limited daily activities (mean = 56, SD = 45), social interactions remained generally preserved (mean = 64, SD = 30). *Supplemental* *Table* provides a detailed overview of all QoL data.

Supplementary Data S4

*MRI acquisition*

Brain MRI with T1-weighted sequences showed isolated vermis atrophy and global cerebellar atrophy (both hemispheric and vermis) in eight and six patients, respectively. Mild global cerebral atrophy was observed in four patients. Two patients had normal brain MRI. No white matter alterations were documented by T2-FLAIR sequence.

Supplementary Data S5

*Sedative-hypnotics use*

Eleven patients (69%) reported using of sedative-hypnotics. Three patients (19%) were taking sedative-hypnotics for insomnia. In particular, two patients took low dosages of BDZ (triazolam or etizolam) and one patient was treated with mirtazapine. Notably, the patient taking mirtazapine did not reported RLS symptoms. Seven patients (44%) used gabapentinoids to improve neuropathic discomfort. Finally, one patient was taking clomipramine for depression and this patient experienced RBD which was likely secondary to the use of this medication.

| **Supplemental Table. Demographic and clinical characteristics of patients with RFC1-spectrum disorders.** | | |
| --- | --- | --- |
|  | **PATIENTS (n° = 16)** | |
|  | **Mean (SD)** | **n° patients (%)** |
| Age (years) | 64 (6) |  |
| Sex (male) |  | 9 (69) |
| Body mass index (kg/m2) | 25 (3) |  |
| Neck circumference (cm) | 39 (3) |  |
| Disease course (years) | 15 (6) |  |
| Motor disability (SARA) | 6 (5) |  |
| Use of sedative-hypnotics° |  | 10 (63) |
| Ferritin levels ng/ml | 135 (31) |  |
| **Clinical manifestation** | |  |
| Cerebellar ataxia |  | 14 (86) |
| Sensitive axonal neuropathy |  | 16 (100) |
| Vestibulopathy |  | 13 (81) |
| Dysautonomia |  | 10 (63) |
| Chronic cough |  | 16 (100) |
| Dysphagia |  | 5 (31) |
| Dysarthria |  | 8 (50) |
| **HRQoL (SF-36)** |  |  |
| Physical functioning | 60 (31) |  |
| Role limitations due to physical problems | 39 (41) |  |
| Bodily pain | 58 (31) |  |
| General health | 37 (24) |  |
| Vitality | 48 (24) |  |
| Social functioning | 64 (30) |  |
| Role limitations due to emotional health problems | 56 (45) |  |
| Emotional well-being | 56 (26) |  |
| **BDI-SF (score ≥ 13)** | 8 (7) | 4 (25) |
| **SAS (raw score ≥ 40)** | 39 (7) | 6 (38) |
| **Brain MRI data** |  |  |
| Cerebellar vermis atrophy |  | 8 (50) |
| Global cerebellar atrophy |  | 6 (38) |
| Data are mean (SD) or percentage. Abbreviations: SARA: Scale for the Assessment and Rating of Ataxia; HRQoL SF-36: health-related quality of life with 3-item Short Form Health Survey; BDI-SF: Beck Depression Inventory Short Form; SAS: Zung Self-Rating Anxiety Scale; MRI: magnetic resonance imaging.  **°**Sedative-hypnotics include one or combination of the following drugs: benzodiazepine/z-drugs, gabapentinoids, sedative antidepressants. | | |

**eReferences**

1. Cortese A, Simone R, Sullivan R, et al. Biallelic expansion of an intronic repeat in RFC1 is a common cause of late-onset ataxia. *Nat Genet*. 2019;51(4):649-658. doi:10.1038/s41588-019-0372-4

2. Schmitz-Hübsch T, du Montcel ST, Baliko L, et al. Scale for the assessment and rating of ataxia: development of a new clinical scale. *Neurology*. 2006;66(11):1717-1720. doi:10.1212/01.wnl.0000219042.60538.92

3. Brazier JE, Harper R, Jones NM, et al. Validating the SF-36 health survey questionnaire: new outcome measure for primary care. *BMJ*. 1992;305(6846):160-164. doi:10.1136/bmj.305.6846.160

4. Dunstan DA, Scott N. Norms for Zung’s Self-rating Anxiety Scale. *BMC Psychiatry*. 2020;20(1):90. doi:10.1186/s12888-019-2427-6

5. Furlanetto LM, Mendlowicz MV, Romildo Bueno J. The validity of the Beck Depression Inventory-Short Form as a screening and diagnostic instrument for moderate and severe depression in medical inpatients. *J Affect Disord*. 2005;86(1):87-91. doi:10.1016/j.jad.2004.12.011

6. Buysse DJ, Reynolds CF, Monk TH, Berman SR, Kupfer DJ. The Pittsburgh Sleep Quality Index: a new instrument for psychiatric practice and research. *Psychiatry Res*. 1989;28(2):193-213. doi:10.1016/0165-1781(89)90047-4

7. Johns MW. A new method for measuring daytime sleepiness: the Epworth sleepiness scale. *Sleep*. 1991;14(6):540-545. doi:10.1093/sleep/14.6.540

8. Morin CM, Belleville G, Bélanger L, Ivers H. The Insomnia Severity Index: psychometric indicators to detect insomnia cases and evaluate treatment response. *Sleep*. 2011;34(5):601-608. doi:10.1093/sleep/34.5.601

9. Allen RP, Picchietti DL, Garcia-Borreguero D, et al. Restless legs syndrome/Willis-Ekbom disease diagnostic criteria: updated International Restless Legs Syndrome Study Group (IRLSSG) consensus criteria--history, rationale, description, and significance. *Sleep Med*. 2014;15(8):860-873. doi:10.1016/j.sleep.2014.03.025

10. Sharon D, Allen RP, Martinez-Martin P, et al. Validation of the self-administered version of the international Restless Legs Syndrome study group severity rating scale - The sIRLS. *Sleep Med*. 2019;54:94-100. doi:10.1016/j.sleep.2018.10.014

11. Stiasny-Kolster K, Mayer G, Schäfer S, Möller JC, Heinzel-Gutenbrunner M, Oertel WH. The REM sleep behavior disorder screening questionnaire--a new diagnostic instrument. *Mov Disord Off J Mov Disord Soc*. 2007;22(16):2386-2393. doi:10.1002/mds.21740

12. American Academy of Sleep Medicine Guidelines | AASM. American Academy of Sleep Medicine – Association for Sleep Clinicians and Researchers. Accessed December 26, 2024. https://aasm.org/clinical-resources/practice-standards/practice-guidelines/

13. Montplaisir J, Gagnon JF, Fantini ML, et al. Polysomnographic diagnosis of idiopathic REM sleep behavior disorder. *Mov Disord Off J Mov Disord Soc*. 2010;25(13):2044-2051. doi:10.1002/mds.23257

14. Sateia MJ. International classification of sleep disorders-third edition: highlights and modifications. *Chest*. 2014;146(5):1387-1394. doi:10.1378/chest.14-0970
